# Conceptualising population health: from mechanistic thinking to complexity science

**DOI:** 10.1186/1742-7622-8-2

**Published:** 2011-01-20

**Authors:** Saroj Jayasinghe

**Affiliations:** 1 Department of Clinical Medicine, Faculty of Medicine, University of Colombo, Kynsey Road, Colombo 8, Sri Lanka

## Abstract

The mechanistic interpretation of reality can be traced to the influential work by René Descartes and Sir Isaac Newton. Their theories were able to accurately predict most physical phenomena relating to motion, optics and gravity. This paradigm had at least three principles and approaches: reductionism, linearity and hierarchy. These ideas appear to have influenced social scientists and the discourse on population health. In contrast, Complexity Science takes a more holistic view of systems. It views natural systems as being 'open', with fuzzy borders, constantly adapting to cope with pressures from the environment. These are called Complex Adaptive Systems (CAS). The sub-systems within it lack stable hierarchies, and the roles of agency keep changing. The interactions with the environment and among sub-systems are non-linear interactions and lead to self-organisation and emergent properties. Theoretical frameworks such as epi+demos+cracy and the ecosocial approach to health have implicitly used some of these concepts of interacting dynamic sub-systems. Using Complexity Science we can view population health outcomes as an emergent property of CAS, which has numerous dynamic non-linear interactions among its interconnected sub-systems or agents. In order to appreciate these sub-systems and determinants, one should acquire a basic knowledge of diverse disciplines and interact with experts from different disciplines. Strategies to improve health should be multi-pronged, and take into account the diversity of actors, determinants and contexts. The dynamic nature of the system requires that the interventions are constantly monitored to provide early feedback to a flexible system that takes quick corrections.

## Introduction

Population health is defined as 'the health outcomes of a group of individuals, including the distribution of such outcomes within the group' [1]. The approach in population health is to improve the health of an entire population and goes beyond the individual focus in medicine or preventive health. This paper proposes that the discourse in population health is dominated by a Newtonian mechanistic view, and there is much to gain by embracing concepts in Complexity Science.

### Analysis

We begin with a brief description and analysis of the mechanistic model, its influence on social sciences and on the way we perceive population health. This is followed by an outline of the new paradigm influenced by Complexity Science and an exploration as to how it sheds light on our understanding of determinants of health.

#### Mechanistic views in science

The mechanistic interpretation of reality can be traced to the influential work by René Descartes and Sir Isaac Newton. Their explanations and theories predicted with a high degree of accuracy most physical phenomena relating to motion, optics and gravity. This paradigm has at least three principles that are especially relevant to population health: reductionism, linearity and hierarchy [2]. The reductionist approach assumes that the whole system (or the macroscopic properties) can be understood by identifying, describing and analysing all its constituent parts (i.e. its microscopic components). The often quoted example is the unlocking of a clock's mechanism by examining its constituent parts. A linear system has two features: proportionality (i.e. the output changes in proportion to the input or a straight-line relationship with the input); and superposition (i.e. the effects of the combined action of different inputs can be figured out and predicted by dissecting the input-output relationships of the individual components) [3]. The overall output is a summation of the constituent parts, and the components of a linear system literally "add up" - there are no surprises or anomalous behaviours. Linearity therefore expects a known input to repeatedly produce a similar effect. Hierarchy suggests a situation with central control or source of power. The energy or power flows from the central source towards the relevant other parts of the system (e.g. the energy from the battery of an electric watch flowing to other areas). An analogous situation is attempted in rigidly structured organisations, in which the top management has centralised control with accountabilities, responsibilities and power percolating down through a system of rules and regulations [4].

#### Mechanistic views in social sciences

It is likely that the Newtonian theories of gravity, optics and motion encouraged mechanistic conceptualisation even in the natural and social sciences. For example, Max Weber stated that "one can, in principle, master all things by calculation" [5]. This reflected his view of an orderly mechanistic world view that was calculable and predicable. Marx too analysed social transformations through a series of predictable linear class struggles. The foundations for the classes were the ownership of means of production (i.e. hierarchy of structuring) and social progress as believed to jump through a series of class struggles between antagonistic social classes (e.g. the workers or proletariat versus the bourgeoisie or upper ruling classes) until a classless communist society was achieved. This pathway of social transformation was considered to be inevitable.

#### Elements of the Newtonian approach in determinants of population health

Some of the previous and current discourses on determinants approach to population health appear to implicitly and explicitly accept a mechanistic view of reality (i.e. reductionism, linearity and hierarchy). Three such examples are described below.

The first is Durkheim's hypothesis that suicides are a product of social influence [6]. His analysis and interpretation showed that rates of suicide reflected social structures that went beyond individual psychological circumstances. Higher rates of suicide in certain communities (in contrast to others) were explained by the relationship between the individual and the moral community. Using variations in these relationships, he proposed three types of suicide: altruistic suicides (i.e. individuals are under intense social control and commit suicide as a matter of honour or duty), egoistical suicide (where the collective acts of society are weak and there is lack of social integration, leading to individuals taking their lives, almost as an act of defiant independence) and anomic suicides (when unrealistic goals set by people, and people's detachment from attainable societal norms, lead to situations in which they cannot cope with life stresses). The hypotheses of Durkheim can be considered as examples of linearity: variations in two interacting factors (i.e. the individual and the moral community) that explain three types of suicide. They also illustrate reductionism: a complex social phenomenon is being telescoped to two main 'agents' in order to explain different outcomes. This does not completely negate the usefulness of such analyses, but indicates that one must be cautious when applying such hypotheses to different social environments and be prepared to accept wide variations in associated features and outcomes.

The second example is the explanations given for the findings in the Black Report of 1980. The Report compiled data from the 1950s and 1970s and showed that life span and morbidity rates from non-communicable diseases were strongly related to measures of social and economic position, which were termed 'social class' [7]. Further exploration of the Whitehall studies of British civil servants revealed the stepwise nature of health inequalities, whereby with each drop in the social status, there was a higher risk of coronary artery disease mortality. This became recognised as the social gradient in health outcomes, in contrast to a dichotomous threshold effect in which the poor have worse indices than the better off [8]. The social gradient was explained using three pathways: (a) low income and its material consequences; (b) a 'cultural-behavioural' explanation in which low-income groups shared a culture that promoted health-damaging behaviours (e.g. acceptance of tobacco smoking as a social norm); and (c) those who were ill or diseased were 'selected' to find themselves in the lower socioeconomic groups, analogous to Darwinian natural selection. This process could operate in the opposite direction too, with the more capable and intelligent moving towards a higher social class. These explanations imply a hierarchy of factors (e.g. health-damaging behaviour) and linearity of outcomes, (e.g. shared cultures promoted adverse health behaviours) and a reductionist approach by attempting to dissect and identify individual factors that are responsible for outcomes.

The third example is from more recent studies that reported disparities in rates of non-communicable diseases (NCDs), communicable diseases and injuries according to social status or socio-economic status or occupations [9]. Several hypotheses are advanced to explain these health inequalities that entail linearity, hierarchy and reductionism. These include:

a) the psychosocial theory: the psychological stresses experienced at home and work place as a result of low social status lead to higher morbidity and mortality rates, predominantly through the autonomic nervous system and hypothalamo-pituitary-adrenal axis;

b) Neo-materialists' explanations: empirical evidence suggests that countries with narrower income inequalities provide easier access to public health, education, and social support. As a result these countries have less social exclusion and narrower disparities in health outcomes;

c) Life course explanations: adverse health influences commencing from foetal life (e.g. from poor maternal nutrition leading to growth retardation), through infancy, childhood, adolescence and young adulthood lead to higher rates of NCDs in later adult life and in the elderly.

A feature of Durkheim's hypothesis and the Black Report is that they cross disciplinary boundaries. For example, Durkheim attempted to explain an epidemiological finding on suicide rates using psychological and social factors, while the Black Report dwells into social class and behavioural science to partly explain the social stratification of illness. The more recent psychosocial theory, neo-materialists' explanations and life course approaches have further increased the interactions with other disciplines. For example, the psychosocial theory incorporates studies from sociology (social status), psychology, immunology and neuro-endocrinology (i.e. the stress pathway) and support from primate research [10]. Although these are healthy deviations from pure reductionism and a hierarchy of contributing factors, features of Newtonian mechanistic views remain. Research is often directed at investigating single or multiple risk factors of diseases and their proximate or distal determinants. Statistical models and epidemiological analysis is extensively used in this discourse, in which linearity is a fundamental assumption to predict future health outcomes. Hierarchy also forms a part of the discourse. Although less visible than reductionism and linearity, it is seen during analyses involving hierarchical modelling and when a strict process of prioritisation or classification is applied to population health policies.

These mechanistic explanations are convenient for conceptualisation and to plan intervention policies, though they are not necessarily a true reflection of reality. Examples include the prescriptive reports by the World Bank and the UK's Department of Health programme for action on health inequalities, and the more pragmatic and flexible Report of the WHO's Commission on Social Determinants of Health [11-13]. In its recommendations, the World Bank advocated several measures that assumed linearity, for example, that economic growth would have a trickle-down effect on health improvement at the household levels [13]. Its recommendations were based on principles of reductionism, i.e. quantifying the burden of illness using Disability Adjusted Life Years, which used a single index to quantify human suffering from different causes. In contrast, the WHO's Commission on Social Determinants of Health has used a more pragmatic approach. It emphasises contextual factors in explaining health inequalities such as governance, macroeconomic policies, social policies, public policies, culture and societal values [11,14]. The broad recommendations (i.e. improving daily living conditions, tackling the inequitable distribution of power, money and resources, and measuring and understanding the problem and assessing the impact of action) are supplemented with numerous examples and success stories from local studies.

The next section gives an overview of Complexity Science and discusses its potential impact on our understanding of population health.

### Complexity Science: a brief overview

The origins of complexity science can be traced to the Systems Theory that was developed by von Bertalanffy in 1920s [15,16]. He noted that biological systems are 'open', in that they are much more influenced by the environment and have to interact with it and exchange matter and energy in order to stay alive. This is in contrast to a material planetary system, which can be considered to be a 'closed system' and perceived to have negligible external influences. A close relation of systems theory is Cybernetics, which studies and explains the autonomy and apparent stability of systems by means of simple circular coupling of events through positive and negative feedback loops. Thus, events originating from the environment that lead to perturbations of the systems are compensated by the negative feedback loops, and the system maintains a 'preferred' state of affairs [3]. An example of an open system is a plant that is dependent on oxygen, sunlight and nutrients in order to produce its energy requirements to grow and reproduce. All these components are obtained from the environment, and interact with each other and with the plant to give other emergent properties such as growth.

Complexity Science that emerged in the 20th century integrates some of the ideas in Systems Theory and Cybernetics. Most natural systems are viewed as consisting of a number of semi-autonomous sub-systems. This idea is implicit in the controversial Gaia Hypothesis, which suggests that the emerging properties of the biosphere are closely related to, and integrated with, the physical components of the earth, such as the atmosphere, the ice caps, the hydrosphere, and the rigid outer layer of the earth [17]. Analogously at the level of a cell, organelles such as the nucleus, cell membrane and mitochondria interact locally with each other, and function as an integrated whole without a central administrator, giving rise to complex behaviours [18].

There are several key ideas in Complexity Science that deviate from the mechanistic approach. These include adaptation, lack of hierarchies, self-organisation, and emergence [4,16,18-20]. Some of the complex systems exhibit adaptation that allow them to modify their structures in order to cope with forces or influences from the environment. These systems are not passive and adapt to the environment by a process of reorganisation, depending on the feedback they receive from within and outside the system. Therefore, such systems are known as complex adaptive systems (CAS), defined as "a collection of individual agents with freedom to act in ways that are not always totally predictable, and whose actions are interconnected so that one agent's action changes the context for other agents" [4]. For population health outcomes (for example, from climate change) the relevant agents could appear to be as unrelated to each other as the cabinet of ministers in an industrialised country is to rural poverty in Asia. Thus, the cabinet of ministers in industrialised countries could take decisions that impact on pollution emissions leading to global warming and climate change. An example is the refusal of some nations to ratify the Kyoto Protocol to reduce greenhouse gas emissions, thus fuelling global warming. In contrast, economic deprivation pushes people in Asia to illegally log forests and use slash-and-burn methods to cultivate cash crops. The latter (for example, in Borneo) leads to a smoke-filled haze covering millions of square kilometres that traps heat.

CAS also have a multi-level 'heterarchical' set of inter-relations, rather than a hierarchical mode of control [16]. Thus, pathways of control flow from multiple agents (e.g. from political decisions in the industrial countries, and loggers setting fire to forests) and change with time. There are numerous local feedback loops linking the interacting agents: the system adapts to changes in the internal and external environment. It is also non-linear (e.g. the temperature rise from industrial emissions and the haze may follow non-linear dynamics) and violates the principles of proportionality and superposition of mechanistic systems.

The boundary between CAS (e.g. a social group) and environment is indistinct and fuzzy, and the two overlap. A particular socio-economic group would have so many characteristics that overlap with other groups, be it wealth, income, class or country of origin, that it is almost impossible to compartmentalise them into distinct groups. The margins also change constantly (i.e. they are dynamic), as people enter or leave the particular group depending on their changing income levels or wealth or perceptions of class.

CAS also show self-organisation and emergence that arise from interactions between sub-systems and the environment [21]. An example is the emergence of complicated colony structures of termites as a result of interactions among termites. These interactions are governed by a few simple rules, and the observable outcome (the termite colony) is more than merely the sum of parts.

### Recent trends in conceptualising population health

Social epidemiologists who explore population health and health inequalities have in recent years moved closer to some of the concepts in Complexity Science. This is suggested by two recent theoretical frameworks that have attempted to explore explanations (as epi+demos+cracy) and life-course approaches (as the ecosocial approach) to health [22,23]. They attempt to integrate among other viewpoints, the social, biological, ecological and historical perspectives [23]. The nature of the analysis goes beyond mere re-interpretation of factors described in one framework (for example, a biological feature such as disparities in disease prevalence) in terms of another framework (such as social class). As described by Nancy Krieger, there are a minimum of four ecosocial constructs in their analyses [23]:

a) Embodiment: how we incorporate socio-physical environmental exposures, susceptibilities and resistances into our biological body. This process begins from our genes, uterine growth and continues throughout the life-course till death, accumulating, neutralising or discarding biological characteristics depending on the complex interplay between the environment and the body. There is acceptance of an 'open' system with multiple levels of analysis of sub-systems and temporal scales. Embodiment could be taken as a form of adaptation of the system to environmental pressures;

b) Pathways of embodiment: these are structured by the societal arrangement of power and resources and constrained by biological characteristics we have gained through evolutions and individual histories. This also denotes how an 'open' system evolves through time, constantly adapting to the dynamic environment;

c) Cumulative interplay between exposure, susceptibility and resistance: there exist multiple levels of sub-systems or factors, and their distributions from sub-cellular levels, going through levels such as individual, community, social group, country and global. These sub-systems change and are dynamic. They interact across a wide range of time scales from nanoseconds (in the case of sub-cellular or biochemical reactions) to millions of years that are relevant time-scales in human evolution;

d) Accountability and agency accepts the reality of multiple roles played by agents, and varying causal explanations at different scales of time and space.

These constructs are analogous to features of a complex system: 'open' systems constantly adapting to the environment, fuzzy borders, lacking clear-cut hierarchies, and non-reductionist in approach.

### Would the discourse on population health gain by using concepts of Complexity Science?

How can we explicitly incorporate principles of Complexity Science to augment these fresh trends in thinking? Firstly, Complexity Science would view population health outcomes in the context of an 'open' system. The patterns of population health outcomes are an emergent property of the system. They arise from a web of causations that result from interactions among dynamic sets of interconnected systems. The interconnecting systems include the political system (e.g. political ideologies in a country, the predominant political governance system), physical environmental factors (e.g. pollution levels, geographic factors, transport), social environment (such as social stratifications, work environment and conditions, and social capital) and biological systems (e.g. genetic predispositions, herd immunity) interacting with each other in a dynamic non-linear fashion [9,11,14,24,25]. This list of systems that influence population health is not exhaustive and will vary according to the context. The manner in which they interact with each other resulting in certain dynamic patterns of population health is less well known. Each context or social group would have its unique combination of factors and processes in the system that determines health outcomes. For example, the health of aboriginal groups in Australia would be heavily influenced by the histories of colonisation, racism, discrimination and marginalisation. These in turn will lead to poverty, disempowerment, social exclusion, and marginalisation. Their cultural beliefs and habits of living in the outback would reinforce the process of marginalisation and alienate them further from urbanised communities. Positive feedback loops could result when marginalisation and isolation heightens potential for further discrimination and stigmatisation by other social groups. Thus, the context matters immensely in understanding the health outcomes of different groups; the lives of aboriginal groups in Australia are literarily and metaphorically a world apart from the lives of civil servants in Whitehall, who would be influenced by the unique hierarchical system, the social status and their behavioural patterns [7,8].

The outcomes of health or inequalities will also have an impact on their own explanatory pathways, and negative and positive feedback loops pervade these relationships [26]. An example of a positive feedback loop is the evidence that social inequalities (in the form of income inequalities) are associated with health inequalities, which in turn promote worsening of income inequalities in a society. The latter is because of a process of selection whereby ill groups who lack social protection become poorer than their healthy counterparts. Evidence suggests that worsening of social inequalities in this manner can lead to a widening of health inequalities [26].

Thus, in order to grasp the essence of complexity of a population one requires at least a basic knowledge of areas as diverse as political science, environmental sciences, biology and historical trajectories, and needs to interact with experts from different disciplines. These disciplines cannot be packaged in separate compartments, but must be incorporated within a wider, almost seamless framework. Such a framework should have no hierarchies. Analogous trends are seen in public health, in which there are calls to abandon use of arbitrary terms such as distal and proximal determinants as this separates factors artificially into two compartments based on spatial or temporal associations with outcomes [27]. In order to operationalise these concepts, newer forms of analyses are required such as multi-level analysis and multi-scale analysis [28,29]. These take into consideration a web or network of causal associations that are dynamic and operating from a number of levels and scales.

Secondly, concepts in Complexity Science can make a significant impact on strategy and policy formulation in relation to health. Assuming population health is an emergent property of a CAS, strategies to improve it should be multi-pronged, work in multiple sectors and at multiple levels. It has to take into account the diversity of factors, determinants and contexts. A systematic review of studies among U.S. minority children to control obesity has shown that strategies with three or more interventions (e.g. nutrition advice, sedentary behaviour reduction, medication) were more effective than those with a smaller number of interventions [30]. The stance taken by the Commission on Social Determinants implicitly accepts this by calling for inter-sectoral action with several successful examples [11]. Because of practical issues these may need to be prioritised, but this is in a way a compromise of addressing complexity. As described before, interactions within a particular system are dynamic and their constant change leads to a range of health outcomes. Theoretically there is no limit to the number of potential interactions and these have to be hypothesised, identified and elucidated.

The third point relates to implementation of interventions to improve population health and management [31]. This would require an organisation structured like a network organisation rather than a hierarchical monolithic structure. Such a networked form, as seen with the modern National Health Service in the UK, allows information to be obtained as it arises and to mobilise responses [2]. If one considers implementation to be a dynamic process, there should be in-built systems to monitor and evaluate progress. Feedback loops are required to enable adjustments to be made with a more accommodating and flexible implementation approach. Since contextual factors are important in determining outcomes, more local innovations and decentralised decision-making should be encouraged at local and community levels. Mechanisms must be in place to constantly monitor progress from the local levels and to provide early feedback to the system for quick correction. Thus, it becomes crucial, if not critical, to invest in information management and communication. Modalities must be in place to facilitate information transfer and exchange. Rapid information transfer alone does not suffice. Policy makers would also have to develop a new mind-set in which they are open to feedback and flexible enough to make changes in strategies and policies quickly. The cyclical process has to continue and be dynamic: policy formulation, implementation, evaluation, feedback and new policies with implementation and corrective action.

Finally, a complexity approach to population health also highlights the contextual nature of factors interacting with each other at a given time. A policy that succeeded in one country during a particular time period may not work in another or at a regional level, because of novel contextual issues and factors or determinants unique to the latter situation. Therefore, one has to be cautious when generalising from the experiences of one location.

## Conclusion

This brief paper suggests that the current discourse on determinants of population health is dominated by a Newtonian mechanistic view. This discourse does not reflect the reality of 'open' complex systems such as population health that are known as Complex Adaptive Systems. Incorporating Complexity Science explicitly to the discourse on population health has important practical implications when conceptualising, strategising, and implementing policies to improve health.

## Competing interests

The author has no financial or non-financial competing interests (political, personal, religious, ideological, academic, intellectual, commercial or any other) to declare in relation to this manuscript.

## Authors' contributions

The author is responsible for conceptualising the issues in the article and writing the manuscript.

## Author's information

The author qualified with a MBBS (Hons) from University of Colombo, and was awarded MD (Colombo), FRCP (London) and MD (Bristol). He is an academic in the University of Colombo, Sri Lanka, a practicing physician at the National Hospital of Sri Lanka, Colombo and a Honorary Research Associate, Department of Epidemiology and Public Health, University College London, UK. He has a special interest in social determinants of health, poverty, health equity, and the application of complexity science to health.
